# Ulcerative and Cytomegalovirus Colitis Associated with Fournier’s Gangrene: A Case Report

**DOI:** 10.34172/mejdd.2024.401

**Published:** 2024-10-30

**Authors:** Ali Berkcan Bozdogan, Gamze Sonmez, Erke Baytok, Goksel Guven, Bengi Ozturk

**Affiliations:** ^1^Department of Internal Medicine, Hacettepe University Faculty of Medicine, Ankara, Turkey; ^2^Hacettepe University, Faculty of Medicine, Ankara, Turkey; ^3^Department of Intensive Care, Hacettepe University Faculty of Medicine, Ankara, Turkey; ^4^Department of Gastroenterology, Hacettepe University Faculty of Medicine, Ankara, Turkey

**Keywords:** Fournier gangrene, Ulcerative colitis, CMV colitis, Complications, Necrotizing fasciitis

## Abstract

Fournier gangrene is a rare but severe complication of ulcerative colitis, characterized by necrotizing fasciitis affecting the genital and perineal regions. We present a case of a 53-year-old man with a history of ulcerative colitis and cytomegalovirus (CMV) colitis who developed Fournier gangrene, an exceptionally uncommon occurrence in this patient population. The patient initially presented with intense pain, swelling, and skin discoloration in the genital area, accompanied by systemic symptoms, including fever. Prompt recognition and intervention are critical due to the aggressive nature of Fournier gangrene, which often results in significant morbidity and mortality. This case underscores the importance of vigilance for unusual presentations of necrotizing infections in patients with inflammatory bowel disease (IBD), particularly those with complicating factors such as immunosuppression and concurrent infections.

## Introduction

 Fournier gangrene is a unique type of necrotizing fasciitis, a condition characterized by deep tissue infection leading to the gradual breakdown of the muscle fascia and the layer of fat beneath the skin. The infection usually advances along the muscle fascia owing to its limited blood circulation, yet muscle tissue often remains unaffected due to its blood flow.^1^ In an epidemiological study conducted in the US, the incidence was 1.6 cases per 100 000 men per year, and the overall mortality was 7.5%.^2^ In the literature, up to 88% mortality rates have been reported in case series.^3^ Factors that increase the likelihood of developing Fournier gangrene include advanced age, diabetes, severe obesity, chronic alcohol abuse, prolonged use of corticosteroids, malignant neoplasms, and HIV infection.^4^ Until now, only a limited number of cases have been documented where Fournier gangrene occurred alongside ulcerative colitis. The predominant bacteria found in Fournier gangrene lesions include both gram-positive and gram-negative bacteria, along with anaerobic bacteria. Occasionally, fungal infections may also be responsible for Fournier gangrene, with Candida spp. or molds being the causative agents, although this is uncommon.^5^ The bacteria responsible for this necrotic infection produce collagenases, facilitating the rapid spread of the infection from the genital area to the front of the abdominal wall and essential organs.^6^

 Fournier gangrene often begins subtly, with 40% of patients showing no initial symptoms, underscoring the importance of early detection for timely intervention. Symptoms typically include intense pain and swelling in the genital or perineal area, fever, and skin discoloration.^6^ In this report, we present a 53-year-old patient diagnosed with ulcerative and cytomegalovirus (CMV) colitis, complicated with Fournier’s gangrene, a highly uncommon complication of ulcerative colitis.

## Case Report

**Figure 1:**
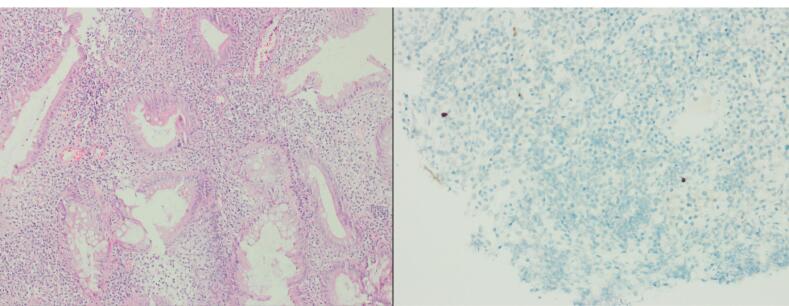
Active colitis is composed of neutrophil leukocytes in the crypt epithelium and stroma. Also, crypt epithelium is showing regenerative and hyperplastic changes ( x 100 magnification, H&E) (left). Immunohistochemically, CMV is nuclear positive in two cells in this section ( x 200 magnification, immunohistochemistry) (right)

**Figure 2:**
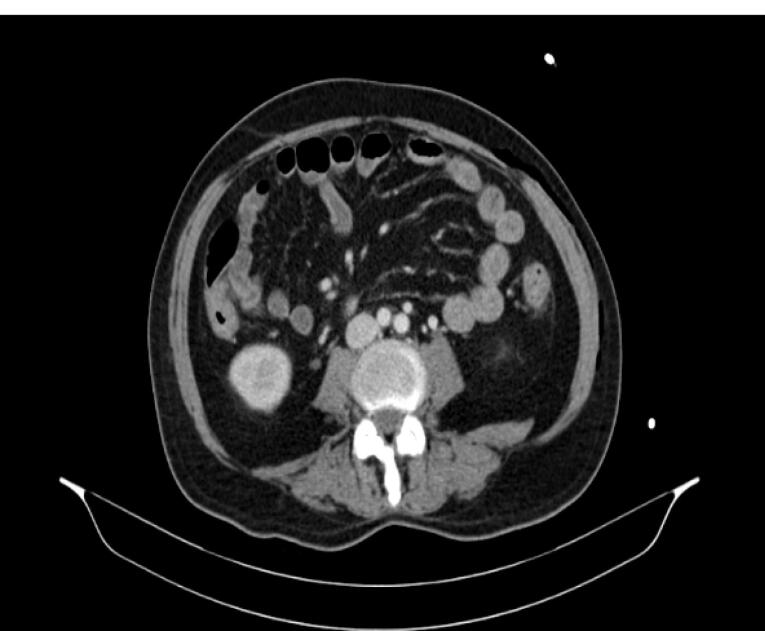
The patient’s abdominal CT scan revealed findings suggestive of intra-abdominal free fluid and mechanical obstruction. Extensive tissue loss was observed in the inguinoscrotal and perianal regions

 In 2009, a 38-year-old male patient was admitted to our hospital due to the red color in the stool. A colonoscopy was performed, and ulcerative colitis was diagnosed. The patient underwent a colonoscopy again in February 2024 due to frequently recurrent diarrhea, and the results of the biopsy obtained from colonoscopy were compatible with CMV colitis in addition to ulcerative colitis (Figure 1). Intravenous (IV) ganciclovir was initiated, and the patient was discharged after approximately 20 days of inpatient treatment. Shortly thereafter, the patient was admitted to the gastroenterology outpatient clinic due to diarrhea lasting 7 days and a discharge lesion in the anal area. The patient was evaluated in the gastroenterology outpatient clinic and subsequently admitted to the internal medicine service due to an exacerbation of ulcerative colitis and the need for IV antibiotic therapy. In the examination, the patient’s body temperature was 39. 4 °C, respiratory rate was 22, pulse rate was 117, and blood pressure was 90/60 mm Hg. The patient was suspected to be dehydrated due to the ulcerative colitis exacerbation, and IV hydration was started. In the follow-up, the patient’s lactate value was 4.0 in blood gas analyses. Due to the patient’s septic condition, treatments with meropenem, teicoplanin, and clindamycin were started. A contrast-enhanced abdominal computed tomography (CT) scan was performed with a prediagnosis of Fournier’s gangrene to investigate the cause of the crepitation. The abdominal CT scan revealed significant diffuse free air and increased inflammatory density in the left gluteal region, perineum, left hemiscrotum, and left lateral abdominal wall, indicating necrotizing fasciitis (Figure 2).

**Figure 3:**
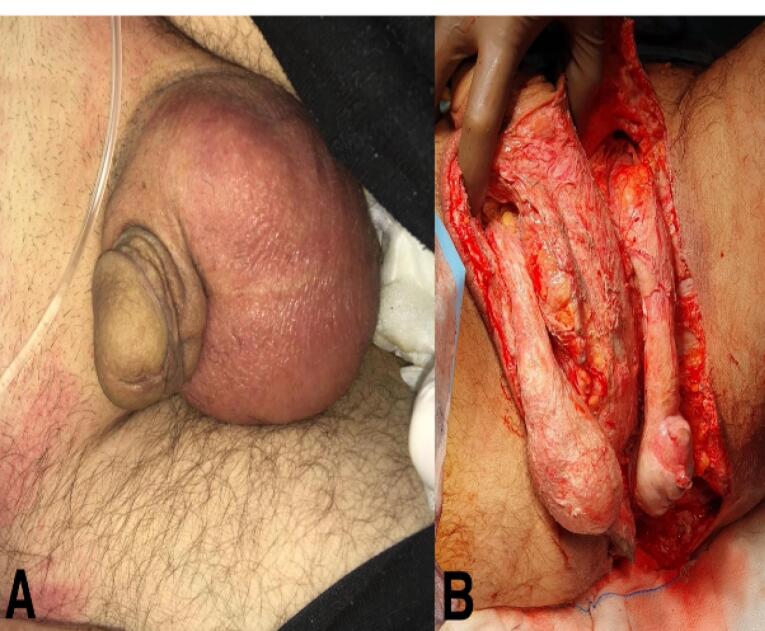
A) Scrotal swelling was seen in the initial presentation B) Removal of damaged or dead tissue from the scrotal and perianal regions through surgery

 The patient required immediate scheduling for perineal debridement, abscess drainage, and drain placement. All necrotic tissue was surgically removed (Figure 3), and pus was collected and sent for culture and sensitivity testing. Based on the test results, treatment with piperacillin/tazobactam, tigecycline, and anidulafungin was initiated. Due to post-operative pain, the patient was not extubated and was admitted to the intensive care unit. During this time, treatment with enoxaparin was initiated due to thrombotic hemorrhagic output from the colostomy. The patient was monitored in the intensive care unit for approximately 2 months. Currently, the patient is being treated with a daily dose of 20 mg of methylprednisolone. Additionally, the Plastic and Reconstructive Surgery Department has planned flap surgery for the patient.

## Discussion

 Fournier’s gangrene is a rapidly advancing type of tissue-decaying infection that affects the outer genital area and frequently extends to the perineum. It is a truly devastating condition with potentially life-threatening consequences. Also, it tends to occur more frequently and with greater severity in individuals with compromised immune systems. Once an entry point for infection is established, various factors can elevate the risk of developing Fournier’s gangrene, with diabetes mellitus being the most prevalent among them. Patients usually exhibit non-specific symptoms early on, resembling cellulitis with redness and hardening of the surrounding area. The onset of necrosis and typical signs of Fournier’s gangrene typically occur later, often leading patients to seek medical attention when severely unwell or experiencing sepsis. Our patient presented with scrotal swelling and discharge in the anal region. Subsequently, he was admitted to the intensive care unit with the preliminary diagnosis of sepsis.

 While treatment modalities have evolved, including surgical debridement and broad-spectrum antibiotics, the prognosis remains guarded, particularly in cases of delayed presentation or underlying comorbidities. Current guidelines advise starting with broad-spectrum antibiotics like carbapenems or piperacillin-tazobactam, along with vancomycin and clindamycin, for empiric treatment to cover both gram-positive and gram-negative bacteria.^7^ However, despite these treatments, the high mortality rate has led to the pursuit of alternative therapies such as hyperbaric oxygen therapy (HBOT). The meta-analysis by Raizandha et al showed that adjunctive HBOT demonstrated a notably reduced mortality rate compared with standard therapy. However, the impact of HBOT on hospital stay duration and the necessity for debridement procedures was not conclusively established.^8^ Following initial treatment, reconstructive surgery is frequently required to address soft tissue defects. Skin grafts, local advancement flaps, scrotal flaps, various types of fasciocutaneous and myocutaneous flaps, and testicular transposition are among the options for reconstructive surgery.^9^

 According to our knowledge, Fournier’s gangrene, occurring as a complication of inflammatory bowel disease (IBD), has been documented in six cases thus far. With few reported cases, there is no specific treatment for IBD complicated by Fournier’s gangrene. Treatment typically includes a series of debridement procedures, administration of broad-spectrum antibiotics, anti-inflammatory therapy for IBD, and, in severe cases, the implementation of diversion colostomy.^10^

## Conclusion

 Fournier gangrene demands heightened clinical vigilance and a coordinated approach to achieve favorable outcomes. Continued research efforts are essential to enhance our understanding of this complex condition and optimize therapeutic strategies, ultimately aiming to improve patient survival and quality of life.
